# Perforation of Meckel's diverticulum caused by a chicken bone: a case report

**DOI:** 10.1186/1752-1947-3-48

**Published:** 2009-02-03

**Authors:** Kim W Chan

**Affiliations:** 1 Department of General Surgery, Royal Blackburn Hospital, Blackburn BB2 3EZ, UK

## Abstract

**Introduction:**

Meckel's diverticulum represents a true diverticulum of the ileum containing all three layers of the bowel wall and is found on the wall of the distal ileum, usually about 2 feet from the ileocaecal valve. Although Meckel's diverticulum is a common congenital abnormality of the gastrointestinal tract, it is often difficult to diagnose. Patients with perforation of Meckel's diverticulum may present with right iliac fossa pain, which mimics acute appendicitis.

**Case presentation:**

A 17-year-old man presented with a 3-day history of lower abdominal pain. On examination, the patient had tenderness in his right iliac fossa. A provisional diagnosis of acute appendicitis was made. The patient was taken to theatre for laparoscopy with the option of appendicectomy. The appendix was found to be normal. An inflamed and perforated Meckel's diverticulum was found to be the cause of the abdominal pain. Meckel's diverticulectomy was performed. The patient made an uneventful recovery and was discharged with further follow-up in the outpatient clinic.

**Conclusion:**

Complications of Meckel's diverticulum can be fatal and early recognition leads to appropriate management. This case report highlights the importance of considering Meckel's diverticulum as a differential diagnosis of acute abdomen in a young patient.

## Introduction

Meckel's diverticulum represents a true diverticulum of the ileum containing all three layers of the bowel wall and is found on the wall of the distal ileum, usually about 2 feet from the ileocaecal valve. It is the vestigial remnant of the omphalomesenteric duct and occurs in approximately 2% of the population, found twice as frequently in men as in women. Only 2% of those individuals who have Meckel's diverticulum are symptomatic and they tend to be typically below the age of two, thereby accounting for why this congenital gastrointestinal anomaly is comparatively better studied in adolescents than in adults.

The complications caused by Meckel's diverticulum include intussusception and volvulus in adolescents and acute bleeding in adults [1]. This is an interesting and unusual case of perforation of Meckel's diverticulum, a very rare complication caused by a chicken bone. To the author's knowledge, and from an extensive review of the literature, such an unusual complication of Meckel's diverticulum has rarely been reported.

## Case presentation

A 17-year-old man presented with a 3-day history of lower abdominal pain. He denied any other symptoms apart from weight loss of 6 kg over the past 2 months. On examination, the patient had tenderness in the right iliac fossa. He had been admitted 2 weeks earlier with rectal bleeding, and was transfused 4 units of red blood cells as his haemoglobin level had dropped to 6.0 g/dL. Gastroscopy and colonoscopy were performed and no abnormalities were found. He was then discharged home with further follow-up in the outpatient clinic.

On this admission, vital signs were within the normal range. Routine blood tests, erect chest and abdominal X-rays were unremarkable. A provisional diagnosis of acute appendicitis was made and initial management included intravenous fluid resuscitation. The presence of peritonism in acute abdomen demands urgent surgical intervention. The patient was consented and taken to theatre for diagnostic laparoscopy with the option of appendicectomy under general anaesthesia.

A normal appendix was identified during laparoscopic examination of the abdomen. An inflammatory mass was seen with turbid fluid collection in the pelvic area on laparoscopy. The inflammatory mass turned out to be a perforated Meckel's diverticulum. The adjacent loop of small bowel was grossly inflamed and dilated. Wedge resection of the perforated Meckel's diverticulum was performed with primary anastomosis of the adjacent small bowel. A small piece of chicken bone was found within the Meckel's diverticulum. The patient made an uneventful recovery postoperatively and was discharged on day 5 with further follow-up in the surgical outpatient clinic.

## Discussion

Meckel's diverticulum was originally described by Fabricius Hildanus in 1598. However, it is named after Johann Friedrich Meckel, who established its embryonic origin in 1809. Although Meckel's diverticulum is a common congenital anomaly of the gastrointestinal tract, it is often difficult to diagnose.

A very small percentage of ingested foreign bodies can cause perforation of the bowel, leading to acute abdomen requiring surgical intervention. Foreign bodies such as dentures, fish bones, chicken bones, toothpicks and cocktail sticks have been known to cause bowel perforation. There are more than 300 cases in the literature of bowel perforation caused by foreign bodies [2]. The majority of patients do not recall ingesting the foreign body, it being discovered either on investigation (abdominal X-ray or computed tomography (CT) scan), or during an operation.

The most common site of perforation is the terminal ileum and colon, although an increased incidence of perforation has been reported in association with Meckel's diverticulum, the appendix and diverticular disease. Perforation of Meckel's diverticulum is usually caused by foreign bodies, a very rare complication as most foreign bodies pass through the gastrointestinal tract without any consequences. Perforation of Meckel's diverticulum caused by a chicken bone has rarely been reported in the literature [3-5].

Perforation of Meckel's diverticulum remains a differential diagnosis of right iliac fossa pain. Meckel's diverticulum is notoriously difficult to diagnose both clinically and radiologically as the symptoms and imaging features are non-specific. The utility of Tc99m-pertechnetate scintigraphy in the diagnosis of ectopic gastric mucosa is well established, particularly in the case of Meckel's diverticulum, despite substantial variation in the reported sensitivity. Radiological as well as clinical findings aid clinicians in diagnosing this congenital anomaly. Perforation of Meckel's diverticulum caused by a chicken bone is a very rare complication and may lead to a fatal outcome if not recognised early.

## Conclusion

Complications of Meckel's diverticulum could be fatal. Early recognition leads to appropriate treatment. This case therefore highlights the importance of considering Meckel's diverticulum as a cause of acute abdomen.

## Competing interests

The author declares that they have no competing interests.

## Authors' contributions

CKW was involved in the conception of the report, literature review, manuscript preparation, editing and submission.

## Consent

Written informed consent was obtained from the patient for publication of this case report and any accompanying images. A copy of the written consent is available for review by the Editor-in-Chief of this journal.
